# The DMH^GABA^ Neurons Play a Crucial Role in the Regulation of Cold‐Induced White Adipose Browning through DMH to LPO Projection

**DOI:** 10.1002/advs.202508513

**Published:** 2025-11-05

**Authors:** Zhijie Su, Yujia Hou, Bingwei Wang, Qian Zhou, Wen Yang, Bingbing Guo, Chenyu Zhang, Miao Zhao, Xiaoning Yang, Jiarui Liu, Xiangyang Xie, Lihua Qin, Weiguang Zhang, Wei L. Shen, Ruimao Zheng

**Affiliations:** ^1^ Department of Anatomy, Histology and Embryology School of Basic Medical Sciences Peking University Beijing 100191 China; ^2^ Basic Medicine Research Innovation Center for Cardiometabolic Diseases Ministry of Education Southwest Medical University Luzhou 646000 China; ^3^ School of Life Science and Technology & Shanghai Clinical Research and Trial Center ShanghaiTech University Shanghai 201210 China; ^4^ Tianjin Medical University/Tianjin Institute of Endocrinology Tianjin 300134 China; ^5^ Neuroscience Research Institute Peking University Beijing 100191 China; ^6^ Key Laboratory for Neuroscience of Ministry of Education Peking University Beijing 100191 China; ^7^ Key Laboratory for Neuroscience of National Health Commission Peking University Beijing 100191 China; ^8^ Beijing Life Science Academy Beijing 102209 China

**Keywords:** cold exposure, dorsomedial hypothalamus, GABAergic neurons, lateral preoptic area, white adipose tissue browning

## Abstract

The browning of white adipose tissue (WAT) can convert energy‐storing WAT into energy‐dissipating beige adipose tissue to combat obesity and metabolic dysfunction. Cold exposure is a WAT browning‐inducing condition in mice. Hypothalamus regulates WAT browning. However, the mechanism of central regulation of WAT browning is still elusive. Here, it is reported that GABAergic neurons in the dorsomedial hypothalamus (DMH^GABA^ neurons) are activated in response to cold exposure. Designer receptors exclusively activated by designer drugs (DREADDs) activation of DMH^GABA^ neurons elicited WAT browning, increased energy expenditure, lowered fat mass weights, and body weight. Conversely, inactivation of DMH^GABA^ neurons showed the opposite effects. Cell type‐specific anterograde tracing revealed that DMH^GABA^ neurons projected to the lateral preoptic area (LPO). Chemogenetic and optogenetic functional circuit interrogation demonstrated that the activation of this projection induced WAT browning. Together, it is uncovered that DMH^GABA^ neurons may act as a key neuronal population controlling cold exposure‐induced WAT browning. DMH^GABA^ neurons regulate WAT browning through DMH to LPO projection. This study expands the understanding of the central mechanism governing the white adipose browning, as well as the anti‐obesity effects of cold therapy.

## Graphical abstract

GABAergic neurons in the dorsomedial hypothalamus (DMH^GABA^ neurons) act as a key neuronal population controlling cold exposure‐induced white adipose tissue browning. DMH^GABA^ neurons regulate the browning through DMH to the lateral preoptic area (LPO) projection. This study expands the understanding of the central mechanism governing the white adipose browning, as well as the anti‐obesity effects of cold therapy.

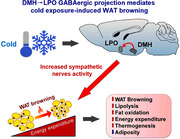

## 1 Introduction

The white adipose tissue (WAT) browning can convert lipid­overloaded WAT into energy‐dissipating beige adipose tissue.^[^ ^1^, ^2^ ^]^ WAT browning is characterized by reduced adipocyte volume, formation of multilocular lipid droplets in white adipocytes, enhanced fat utilization, elevated energy expenditure (EE), reduced whole‐body adiposity, and lowered body weight.^[^ ^1^, ^3^, ^4^ ^]^ It is estimated that in adult humans, the browned subcutaneous fat may account for nearly 15% of resting EE or roughly 550 kcal day^−1^.^[^ ^5^, ^6^ ^]^ Therefore, the promotion of WAT browning has emerged as a promising novel strategy for treating obesity and related metabolic disorders.^[^ ^7^, ^8^ ^]^ However, the mechanism underlying WAT browning induction is still elusive. The WAT browning in mice can be substantially induced by specific conditions,^[^ ^2^, ^9^ ^]^ such as cold exposure.^[^ ^10^, ^11^, ^12^, ^13^ ^]^ However, the mechanisms by which cold exposure induces WAT browning remain partially understood.

The hypothalamus, a primary brain center for metabolic regulation, acts as a key site for integrating the signals of external conditions.^[^ ^14^, ^15^, ^16^, ^17^ ^]^ Of note, the hypothalamus has also been identified as a region involved in controlling WAT browning.^[^ ^13^, ^14^, ^18^, ^19^ ^]^ In recent years, the inherent complexities of the hypothalamic circuits regulating adipose homeostasis have been studied.^[^ ^16^, ^20^, ^21^, ^22^ ^]^ The DMH is a master metabolic regulator in the hypothalamus.^[^ ^23^, ^24^ ^]^ The DMH harbors both GABAergic and glutamatergic neurons, with the former as the dominant population.^[^ ^21^, ^25^ ^]^ Nevertheless, the hypothalamic mechanism underlying the WAT browning‐inducing conditions, such as cold exposure induce WAT browning is still unclear.

In the present study, by applying a canonical WAT browning‐inducing condition, whole‐brain mapping with quantitation of neuronal activation at single‐cell resolution by c‐Fos staining, brain nucleus genome‐wide transcriptomic sequencing analysis, Cre‐reporter fluorescent labeling, Cre‐dependent anterograde neurocircuit viral tracer, and neuron type‐specific designer receptors exclusively activated by designer drugs (DREADDs) and optogenetic approaches, we revealed a previously unrecognized role for the GABAergic neurons in the dorsomedial hypothalamus (DMH^GABA^ neurons) in centrally governing WAT browning induced by cold exposure. We unveiled that DMH^GABA^ neurons project to the lateral preoptic area (LPO), and this circuitry mediates the induction of WAT browning. In summary, we uncovered that DMH^GABA^ neurons act as a key neuronal population controlling WAT browning. DMH^GABA^ neurons regulate cold exposure‐induced WAT browning through GABAergic DMH to LPO projection. These findings provide a novel insight into the mechanism underlying the central regulation of WAT browning.

## 2 Results

### 2.1 DMH Responds to WAT Browning‐Inducing Cold Exposure

**Figure 1:**
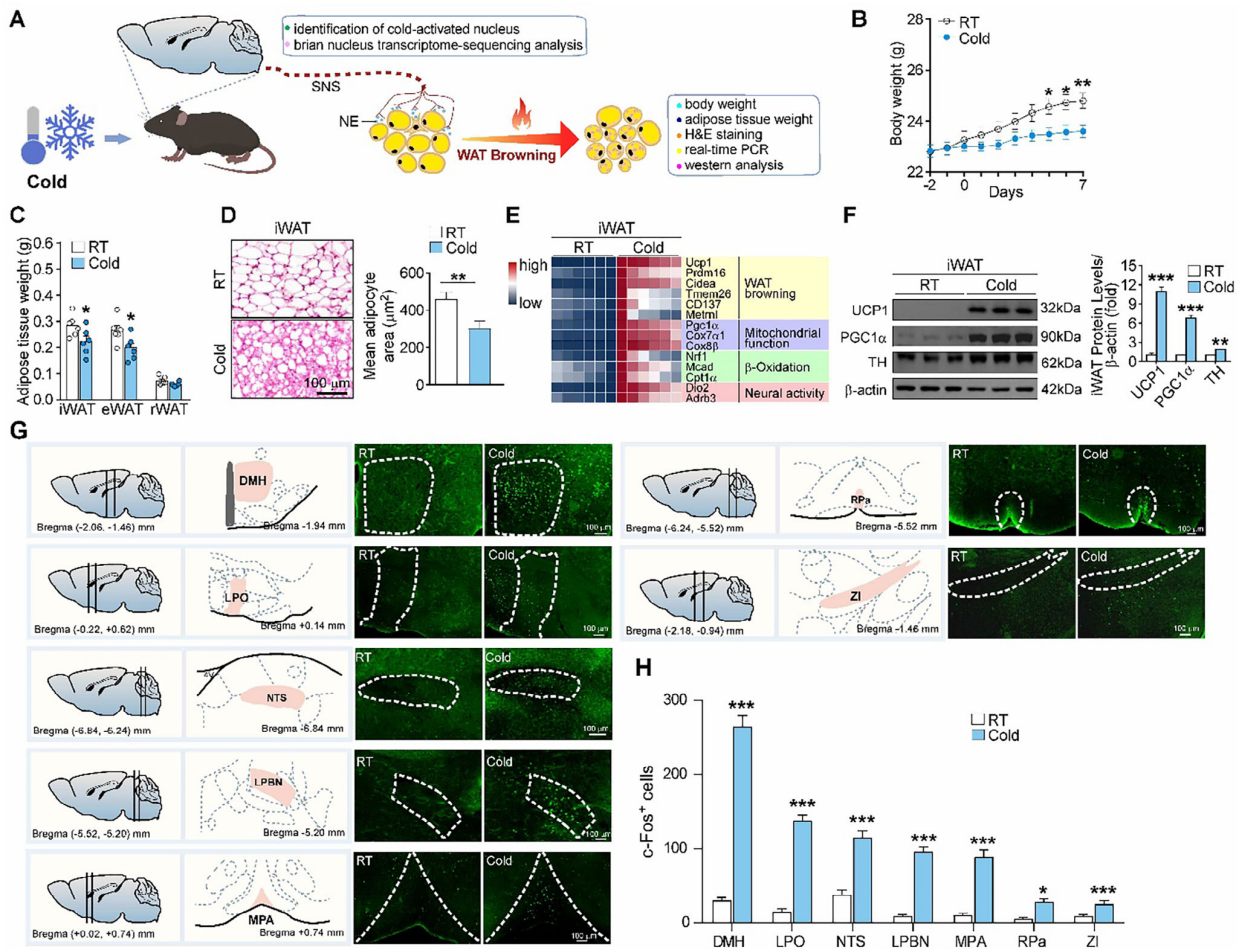
DMH neurons are activated under cold exposure. A) The flow diagram of experimental design for deciphering the neural mechanism underlying the central regulation of WAT browning. B) Body weight changes. C) Weight changes of fat mass. iWAT, inguinal WAT; eWAT, epididymal WAT; rWAT, retroperitoneal WAT. D) Representative images of H&E staining of iWAT sections and the quantification of adipocyte areas. RT, room temperature. E) Heatmap of expression levels of genes involved in WAT browning, mitochondrial function, β‐oxidation, and neural activity in the iWAT; the gene expression levels were quantified by quantitative real‐time PCR analysis. F) Western analysis of UCP1, PGC1α, and TH protein levels in the iWAT. G) Representative images of c‐Fos immunoactivity in the DMH, LPO, NTS, LPBN, MPA, RPa, and ZI under chronic cold exposure. H) The graph depicts quantification of c‐Fos^+^ neurons in different brain regions under cold exposure. Mice were 10‐week‐old males, *n* = 6, biologically independent samples from RT and Cold groups. All data are expressed as means ± SEM. The *P* values are calculated based on unpaired Student's t‐test (C, D, F, and H) and repeated measures two‐way ANOVA with Bonferroni's corrections (B). ^***^ *p* < 0.001, ^**^ *p* < 0.01, ^*^ *p* < 0.05.

To decipher the neural mechanism underlying the central regulation of WAT browning, we initially evaluated the phenotypes of WAT browning elicited by the chronic cold exposure (**Figure** **1**A). Cold exposure increased food intake (Figure S1A, Supporting Information). The body weights were reduced as compared with controls (Figure 1B). The fat mass weights of iWAT (0.22 ± 0.02 g vs 0.28 ± 0.02 g, *p* < 0.05) and epididymal white adipose tissue (eWAT) (0.20 ± 0.04 g vs 0.26 ± 0.03 g, *p* < 0.05), but not rWAT (retroperitoneal WAT), were decreased (Figure 1C). The typical smaller brown‐like adipocytes with multilocular lipid droplets in inguinal WAT (iWAT) were observed (Figure 1D). The expression level of the genes associated with WAT browning (*Ucp1, Prdm16, Cidea, Tmem26, Cd137, and Metrnl*), mitochondrial biogenesis (*Pgc1α, Cox7α1, and Cox8β*), β‐oxidation (*Nrf1, Mcad, and Cpt1α*), and sympathetic nerve activation (*Dio2 and Adrb3*) was upregulated in iWAT (Figure 1E). The protein level of UCP1, PGC1α, and tyrosine hydroxylase (TH) was increased in iWAT (Figure 1F; Figure S1B, Supporting Information). These observations validated that a robust WAT browning could be induced by cold exposure.

To identify the brain nuclei that may respond to the WAT browning‐inducing condition, cold exposure, we systematically performed whole‐brain immunostaining for c‐Fos expression, an indicator of neuronal activation.^[^ ^15^, ^26^ ^]^ Of note, the DMH, the LPO, the nucleus tractus solitarius (NTS), the lateral parabrachial nucleus (LPBN), the medial preoptic area (MPA), the raphe pallidus nucleus (RPa), and the zone incerta (ZI) were activated by cold exposure (Figure 1G,H; Figure S1C, Supporting Information). In particular, the DMH was robustly activated by cold exposure (Figure 1G,H). Previous studies have implicated the DMH in the central regulation of thermogenesis and energy expenditure, potentially including WAT browning.^[^ ^24^, ^27^ ^]^ Together, these findings suggest that the neurons in these nuclei, such as the DMH, may be responsive to cold exposure and involved in the central regulation of WAT browning.

### 2.2 WAT Browning‐Inducing Cold Exposure Activates DMH^GABA^ Neurons

**Figure 2:**
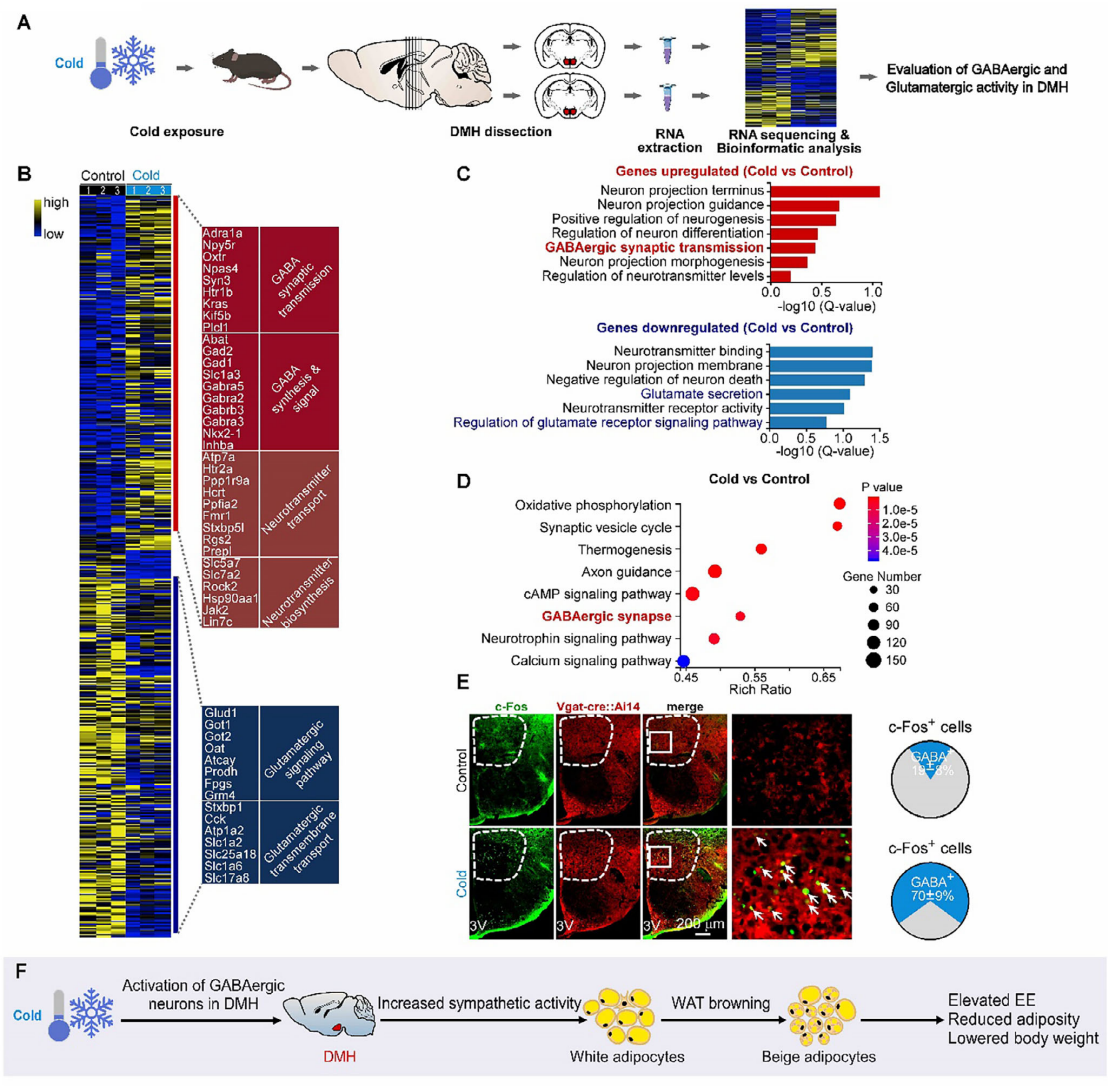
Cold exposure activates GABAergic neurons in the DMH. A) Design for DEG analysis in the DMH by RNA deep sequencing under cold exposure. B) The genes related to neural activity in the DMH are induced by cold exposure. The relative expression levels were color‐coded and indicated in the Figure (upregulated, yellow; downregulated, blue; FDR < 0.05). Rows indicate individual genes, and columns indicate individual mice (*n* =  3 per group). C) GO analysis of DEGs in the DMH. D) KEGG pathway enrichment analysis for the DEGs clusters induced by cold exposure. E) Coronal sections of Vgat‐cre::Ai14 mice subjected to cold exposure and immunostained for c‐Fos. The percentage of double‐positive for c‐Fos (green) and Vgat (red) was shown in the right (*n* = 4 mice per group). White arrows indicate the neurons that were double‐positive for c‐Fos and Vgat. Scale bar: 200 µm. F) Proposed mechanism underlying cold exposure‐induced WAT browning.

To gain a comprehensive assessment of neuronal activation in the DMH under cold exposure, we performed a genome‐wide transcriptomic sequencing analysis (**Figure** **2**A). This analysis identified the differentially expressed genes (DEGs) within the DMH (Figure 2B). These DEGs showed an increased expression level of the genes related to the GABAergic function (Figure 2B). In contrast, the expression level of the genes related to the glutamatergic function was not strikingly changed (Figure 2B). The genes involved in GABA synaptic transmission and GABA synthesis, as well as neurotransmitter function, such as regulation of neurotransmitter transport and biosynthesis, were upregulated in the DMH (Figure 2B). Gene Ontology (GO) analysis revealed an increased function of GABAergic synaptic transmission (Figure 2C). The Kyoto Encyclopedia of Genes and Genomes (KEGG) pathway enrichment analysis also showed that expression of the genes related to GABAergic synapse was enhanced under these conditions (Figure 2D). To determine whether the GABAergic neuronal population in the DMH could certainly be activated under cold exposure, we bred Vgat‐ires‐cre knock‐in mice with Ai14 Cre‐reporter mice to label the GABAergic neurons with the red fluorescent protein, tdTomato. We determined the activated neurons within the DMH by analyzing the colocalization of c‐Fos/tdTomato. We identified that over half of c‐Fos^+^ neurons were GABAergic in the DMH under cold exposure (70 ± 9% vs 19 ± 8%) (Figure 2E). Taken together, these findings revealed that the DMH may be a predominant nucleus that is activated by cold exposure, and the *DMH^GABA^ neurons* may play important roles in the central regulation of WAT browning (Figure 2F).

### 2.3 Inactivation of DMH^GABA^ Neurons Inhibits Cold‐Induced WAT Browning

**Figure 3:**
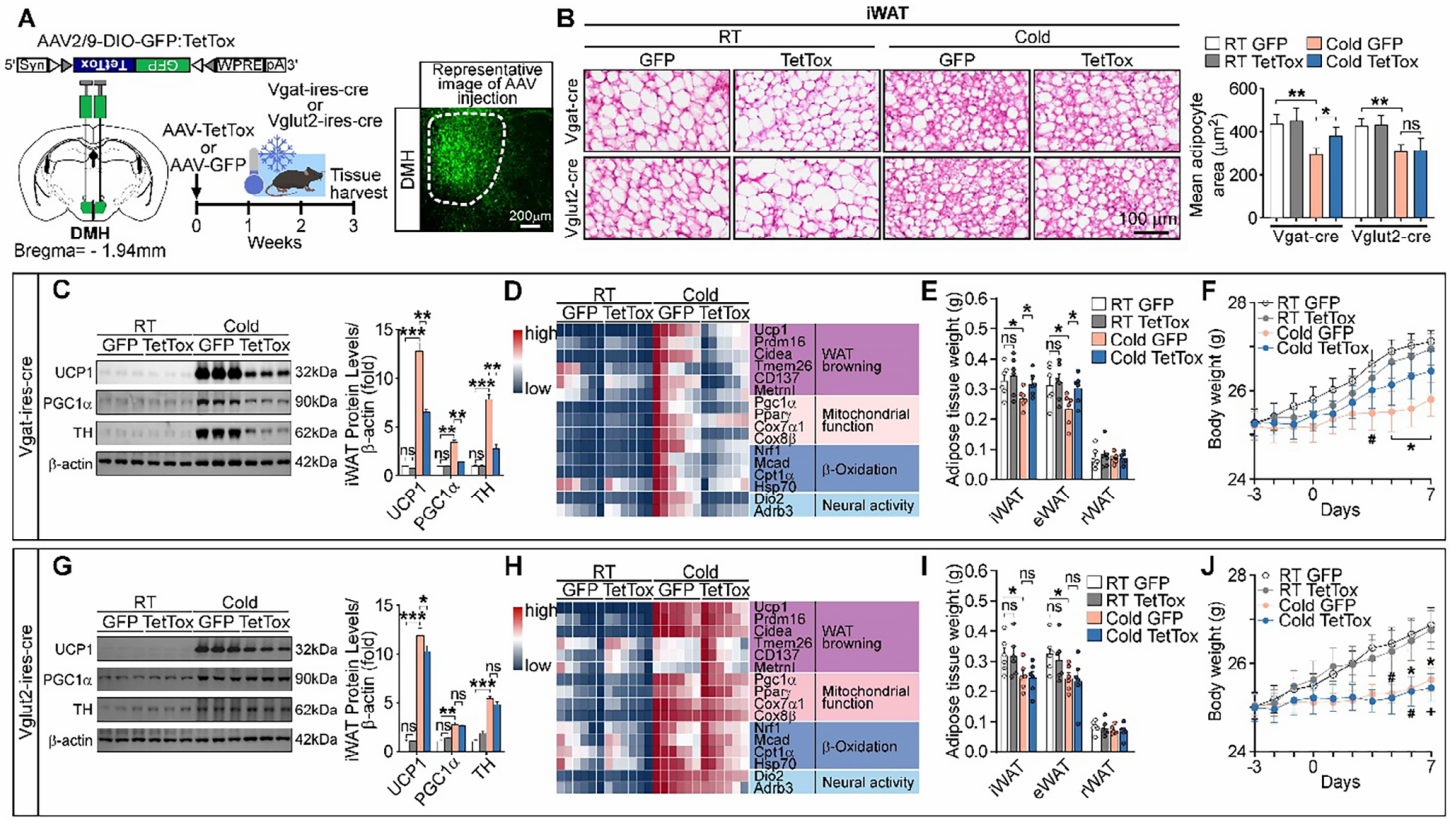
Inhibition of DMH^GABA^ neurons abolishes the adipose browning induced by cold exposure. A) Schematic and representative images illustrated DMH injection of viral neural toxin (AAV‐DIO‐TetTox:GFP, bilateral) into Vgat‐ires‐Cre or Vglut2‐ires‐Cre mice. The AAV‐DIO‐GFP was used as the control. The injected mice were subjected to cold exposure to elicit white adipose browning. B) Representative images of H&E staining of iWAT sections and quantification of adipocyte areas (*n* = 3 mice per group, with 5 or 6 sections per mouse) (C–J). Inactivation of DMH^Vgat^ neurons (C–F), but not DMH^Vglut2^ neurons (G–J), diminished WAT browning phenotypes induced by cold exposure (n =  6 for each group, from two independent experiments). Changes of UCP1, PGC1α, and TH proteins in the iWAT (C) and (G). Heatmap representing expression levels of genes related to WAT browning, mitochondrial function, β‐oxidation, and neural activity in the iWAT (D) and (H). Weight changes of individual fat mass (E) and (I), iWAT; eWAT, epididymal WAT; rWAT, retroperitoneal WAT. Bodyweight changes (F) and (J). All data are presented as means ± SEM. The *p* values are calculated based on ordinary two‐way ANOVA with Tukey's corrections (B, C, E, G, and I), and repeated measures two‐way ANOVA with Bonferroni's corrections (F and J). ns, not significant; ^**^ *p* < 0.01, ^*^ *p* < 0.05, ^++^ *p* < 0.01, ^+^ *p* < 0.05.

As the majority of c‐Fos^+^ neurons in the DMH were GABAergic under cold exposure, we asked whether DMH^GABA^ neurons may play a critical role in the central regulation of WAT browning. We employed a loss‐of‐function strategy by inactivating DMH^GABA^ neurons and DMH glutamatergic neurons (DMH^Vglut2^ neurons) with tetanus toxin (TetTox), respectively (**Figure** **3**A). Following stereotaxic injection of AAV expressing Cre‐dependent TetTox (AAV‐DIO‐GFP: TetTox) into the DMH of Vgat‐ires‐cre mice, the GABAergic neurons in the DMH were permanently inactivated. Of note, inactivation of DMH^GABA^ neurons dampened the white adipose browning phenotypes induced by cold exposure (Figure 3B–D) and diminished the numbers of cold‐induced small‐volume beige adipocytes in iWAT (Figure 3B). The protein level of UCP1, PGC1α, and TH was restored in iWAT (Figure 3C). The expression level of the genes associated with WAT browning (*Ucp1, Prdm16, Cidea, Tmem26, Cd137, and Metrnl*), mitochondrial biosynthesis (*Pgc1α, Cox7α1, and Cox8β*), β‐Oxidation *(Nrf1, Mcad, Cpt1α, and Hsp70)*, and sympathetic neural activity (*Dio2 and Adrb3*) was normalized in iWAT (Figure 3D). Fat mass weights of iWAT and body weight were also normalized, as compared with controls (Figure 3E,F). In addition, inactivation of DMH^Vglut2^ neurons by injection of AAV expressing Cre‐dependent TetTox in the DMH of Vglut2‐ires‐cre mice did not markedly affect cold‐induced WAT browning phenotypes (Figure 3B,G–J). Gene knockout of the vesicular GABA transporter (VGAT, encoded by Vgat, also known as Slc32a1) specifically in DMH neurons to disrupt GABA release also resulted in increased body weight (Figure S2A,B, Supporting Information) and fat mass (Figure S2C, Supporting Information) without affecting food intake (Figure S2D, Supporting Information). In addition, glucose tolerance was impaired (Figure S2E, Supporting Information); locomotor activity was unchanged when compared with the control group (Figure S2F, Supporting Information). Collectively, these findings demonstrated that DMH^GABA^ neurons, rather than DMH^Vglut2^ neurons, are required for cold‐induced white fat browning. The DMH is an essential brain nucleus for WAT browning induction, and the DMH^GABA^ neurons serve as a key neuronal population in regulating WAT browning.

### 2.4 DREADDs Activation of DMH^GABA^ Neurons Elicits WAT Browning

**Figure 4:**
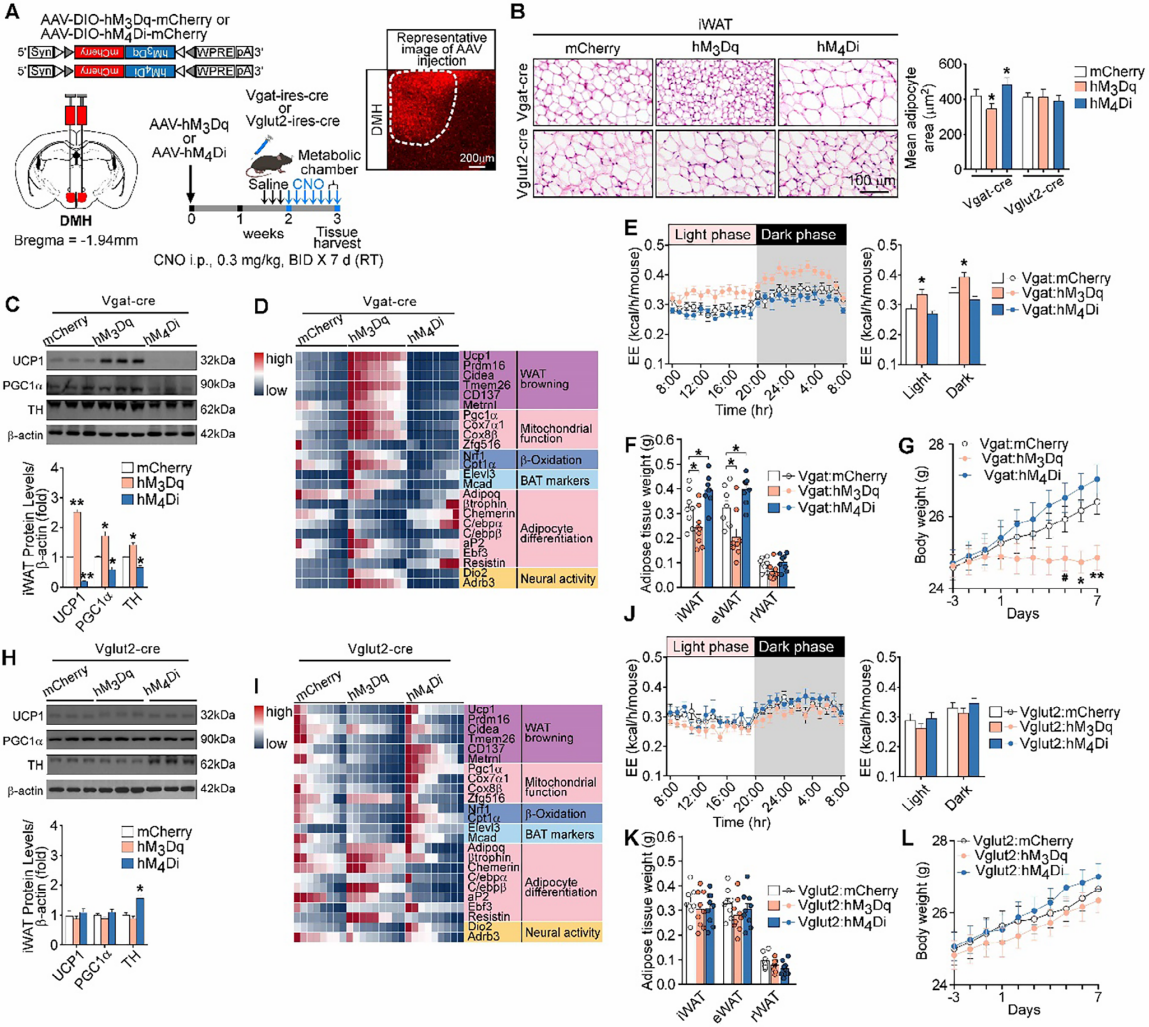
Activation of DMH^GABA^ neurons by DREADDs elicits WAT browning. A) Schematic and representative images demonstrate the bilateral injection of AAV‐DIO‐hM3Dq‐mCherry or AAV‐DIO‐hM4Di‐mCherry (bilateral) into the DMH of Vgat‐ires‐Cre or Vglut2‐ires‐Cre mice. CNO was delivered (i.p.) to activate or inhibit the neurons expressing hM3Dq or hM4Di, respectively. CNO was administered intraperitoneally at 0.3 mg kg^−1^ at RT, twice daily (BID) for seven consecutive days (×7 d). B) Representative images of H&E staining of iWAT and quantification of adipocytes (*n* = 3 mice per group). C–L) Stimulation of DMH GABAergic neurons, but not DMH glutamatergic neurons, enhanced WAT browning phenotypes. Protein levels of UCP1, PGC1α, and TH in the iWAT (C) and (H). Heatmap of expression levels for genes related to WAT browning in the iWAT (D) and (I). Basal EE (E) and (J). Fat mass weights (F) and (K). Body weight (G) and (L). All data are presented as means ± SEM. The *P* values are calculated on the basis of ordinary two‐way ANOVA with Tukey's corrections (B, E, F, J, and K), repeated measures two‐way ANOVA with Bonferroni's corrections (G and L), and unpaired Student's t‐test (N, P, and Q). ^***^ *p* < 0.001, ^**^ *p* < 0.01, ^*^ *p* < 0.05, ^#^ *p* < 0.1.

To test whether activation of DMH^GABA^ neurons may induce WAT browning, the DREADDs approaches were used. The AAVs carrying a Cre‐dependent hM3Dq:mCherry or hM4Di:mCherry were bilaterally injected into the DMH of Vgat‐ires‐Cre or Vglut2‐ires‐Cre mice (**Figure** **4**A). The chemogenetic activation of DMH^GABA^ neurons or DMH^Vglut2^ neurons was achieved by injection of the synthetic ligand clozapine‐N‐oxide (CNO) (Figure 4A; Figure S3A, Supporting Information). Mice received twice‐daily intraperitoneal injections of CNO (0.3 mg kg^−1^) to modulate neural activity for seven days at room temperature. Notably, chemoactivation of DMH^GABA^ neurons caused morphological phenotypes of WAT browning, including decreased cell sizes and increased multilocular lipid droplets in iWAT (Figure 4B), increased expression level of the proteins and genes associated with WAT browning (Figure 4C,D), elevated oxygen consumption (VO_2_) (Figure S3D, Supporting Information) and carbon dioxide production (VCO_2_) (Figure S3D, Supporting Information), increased EE (Figure 4E), decreased respiratory exchange ratio (RER) (Figure S3F, Supporting Information), reduced fat mass weights of iWAT and eWAT (Figure 4F), and lowered body weight (Figure 4G). Additionally, no significant morphological changes were observed in eWAT (Figure S3B, Supporting Information), and food intake and locomotor activity were unchanged (Figure S3C,G,K, Supporting Information). Conversely, chemogenetic inactivation of DMH^GABA^ neurons showed opposite effects in the WAT browning (Figure 4B–G). Chemogenetic activation or inactivation of DMH^Vglut2^ neurons did not alter the phenotypes of WAT browning (Figure 4B,H–L). Together, these findings demonstrate that activation of DMH^GABA^ neurons induces WAT browning, elevates VO_2_ and EE, and reduces adiposity and body weight.

### 2.5 Optogenetic Activation of DMH^GABA^ Neurons Causes WAT Browning

**Figure 5:**
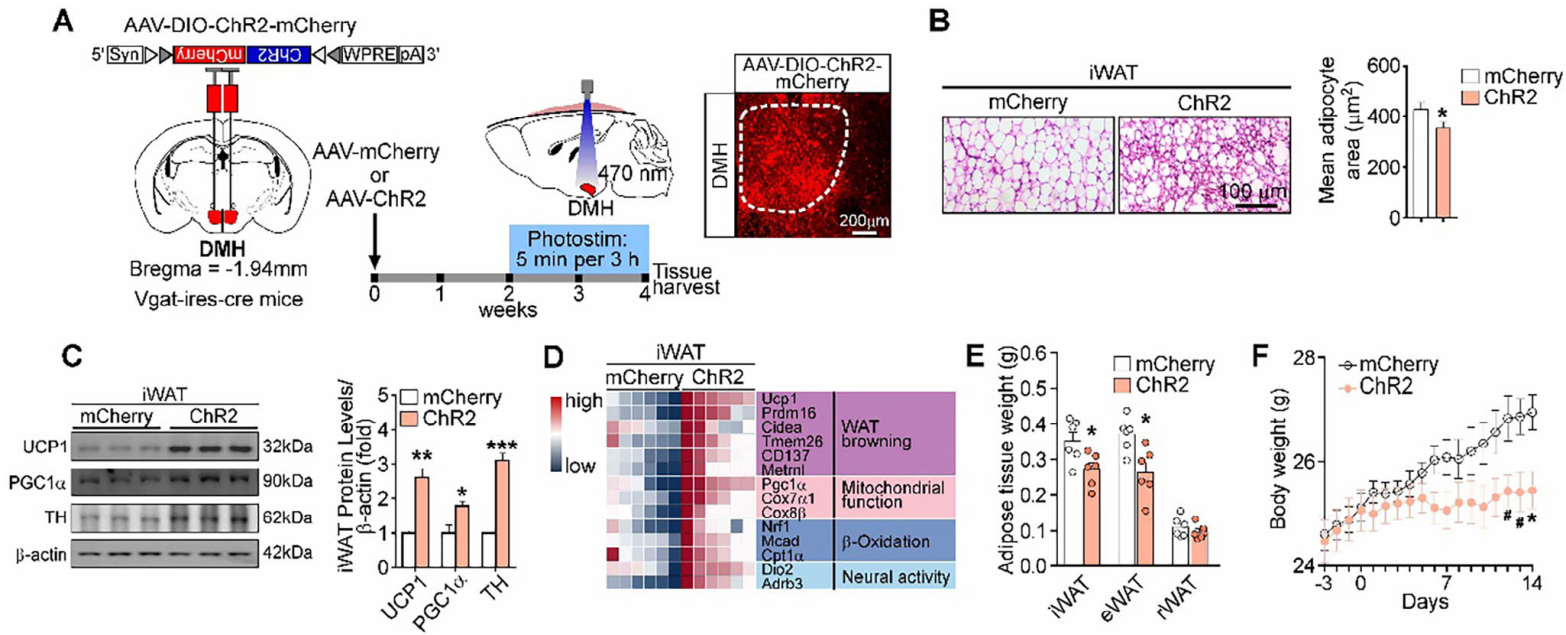
Optogenetic stimulation of DMH^GABA^ neurons elicits WAT browning. A) Scheme for optogenetic stimulation of DMH GABAergic neurons by ChR2. The  white line indicates the positions of optical inserts. Light pattern: 5 min, 20 Hz, for every 3 h, repeated over 14 days. B) Representative images of H&E staining of iWAT and quantification of adipocyte areas (*n* = 3 mice per group). C–F) Optogenetic activation of DMH GABAergic neurons promotes white adipose browning (n = 6 mice per group). Protein levels of UCP1, PGC1α, and TH in the iWAT (C). Heatmap of expression levels of genes related to WAT browning and neural activity in the iWAT (D). Fat mass weights (E). Body weight (F). All data are presented as means ± SEM. The *P* values are calculated on the unpaired Student's t‐test (B and E) and repeated measures two‐way ANOVA with Bonferroni's corrections (F). ^*^ *p* < 0.05, ^#^ *p* < 0.1.

To validate the function of DMH^GABA^ neurons in WAT browning induction, an optogenetic approach was used. We expressed Cre‐dependent ChR2 in the DMH of Vgat‐ires‐Cre mice, and optogenetically activated DMH^GABA^ neurons (**Figure** **5**A). Similar to the chemogenetic activation, optogenetic stimulation of DMH^GABA^ neurons also induced WAT browning phenotypes in iWAT but not in eWAT (Figure 5B; Figure S4A–E, Supporting Information), increased the expression level of proteins and genes associated with WAT browning induction (Figure 5C,D; Figure S4C, Supporting Information), reduced fat mass weights (Figure 5E), and lowered body weight (Figure 5F). Thermal imaging showed that optogenetic stimulation of DMH^GABA^ neurons increased surface temperature of iWAT and BAT, as compared with controls (Figure S4B,C, Supporting Information). Taken together, these findings confirmed that activation of DMH^GABA^ neurons may effectively induce white fat browning. The DMH^GABA^ neurons may be one of the critical neuronal populations for the central regulation of WAT browning induced by external conditions.

### 2.6 DMH^GABA^ Neurons Regulate WAT Browning via a Projection to LPO

**Figure 6:**
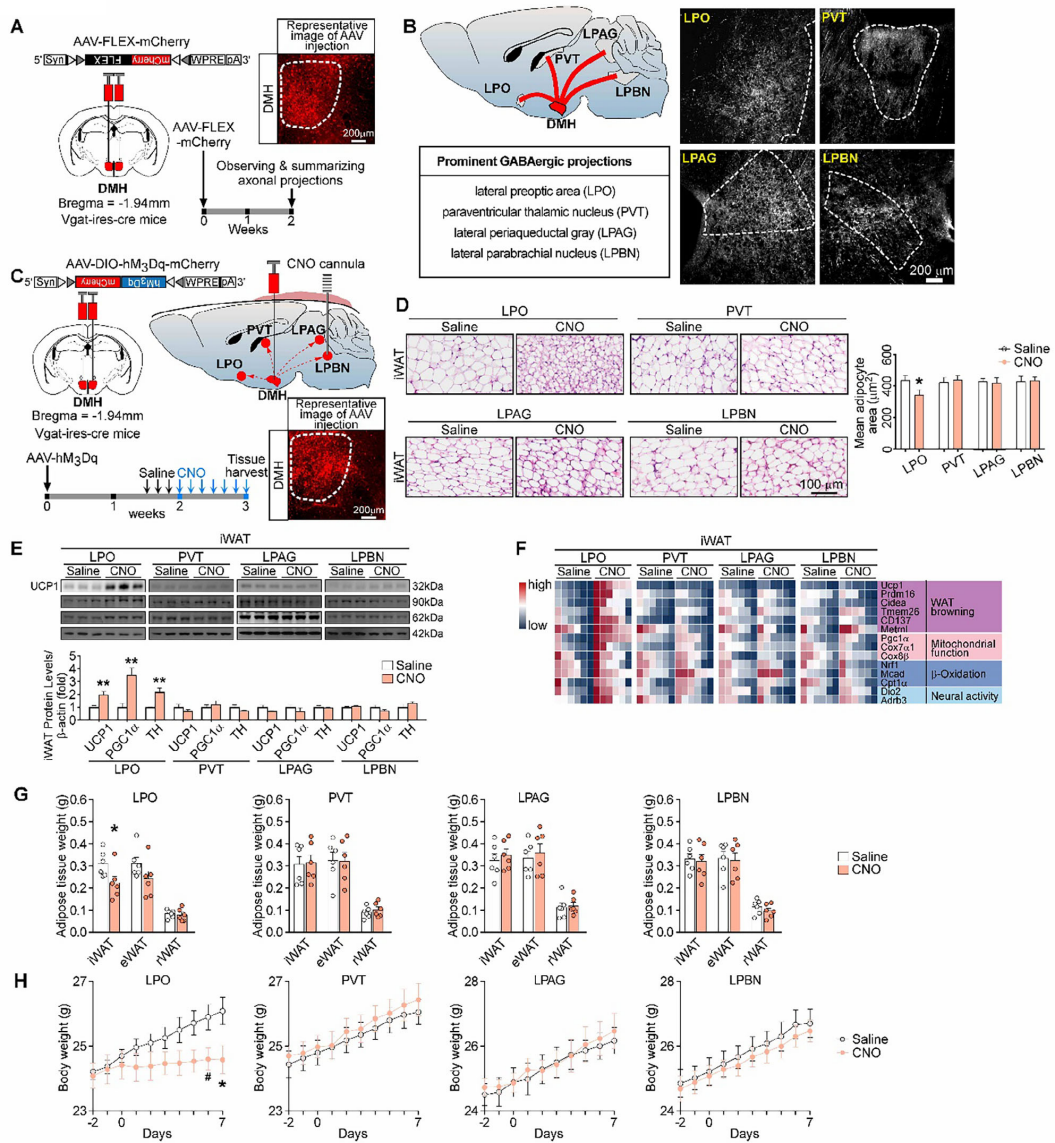
DMH^GABA^ neurons regulate WAT browning via a projection to LPO. A) Design for axonal tracing of DMH GABAergic neurons by using AAV‐FLEX‐mCherry bilaterally injected into the DMH of Vgat‐ires‐Cre mice. B) The whole‐brain axonal projection pattern of DMH GABAergic neurons. LPO, lateral preoptic area; PVT, paraventricular thalamic nucleus; LPAG, lateral periaqueductal gray; LPBN, lateral parabrachial nucleus. Representative image from three independent experiments. Scale bar: 200 µm. C) Diagrams showing chemogenetic stimulation of different axon terminals of DMH GABAergic neurons. The AAV‐DIO‐hM3Dq‐mCherry was injected into the DMH bilaterally, and the cannulae used for CNO infusion were implanted into the LPO, PVT, LPAG, and LPBN as indicated. D) Representative images of H&E staining of iWAT after chemogenetic stimulation of downstream brain regions of DMH GABAergic axon terminals and quantification of adipocyte areas (*n* = 3 mice per group). E,F) Changes in protein levels (E) and gene expression levels (F) related to white adipose browning after chemogenetic stimulation of DMH GABAergic axon terminals in downstream brain regions (*n* =  6 biologically independent samples per group). G) Weight changes of individual fat mass after chemogenetic activation of DMH GABAergic terminals at the LPO, PVT, LPAG, and LPBN (*n* =  6 biologically independent samples per group, unpaired Student's t‐test). The CNO was infused in these indicated brain sites. H) Bodyweight changes after chemogenetic activation of DMH GABAergic terminals at the LPO, PVT, LPAG, and LPBN (n =  6 mice per group). All data are presented as mean ± SEM. Statistical analyses were performed using repeated measures two‐way ANOVA analyses followed by Bonferroni's post hoc test (^*^ *p* < 0.05, ^#^ *p* < 0.1).

To investigate downstream projections of DMH^GABA^ neurons for eliciting WAT browning, we injected a Cre‐dependent anterograde AAV viral tracer (AAV‐FLEX‐ mCherry) into the DMH of Vgat‐ires‐Cre mice (**Figure** **6**A). This strategy revealed prominent projections of DMH^GABA^ neurons to the LPO, the paraventricular thalamic nucleus (PVT), the lateral periaqueductal gray (LPAG), and the lateral parabrachial nucleus (LPBN) (Figure 6B). To examine the roles for axon terminals of DMH^GABA^ neurons in these downstream nuclei, we injected AAV‐DIO‐hM3Dq:mCherry bilaterally into the DMH of Vgat‐ires‐Cre mice (Figure 6C). To activate the axon terminals of the DMH, we implanted cannulae over the LPO, the PVT, the LPAG, and the LPBN, respectively, for infusing CNO (Figure 6C). Notably, we found that activation of axon terminals of DMH^GABA^ neurons in the LPO induced typical phenotypes of WAT browning (Figure 6D–H). Infrared thermal imaging also showed a significant increase in the surface temperature of iWAT and BAT (Figure S5A–C, Supporting Information). Whereas activation of axon terminals of DMH^GABA^ neurons in the PVT, the LPAG, or the LPBN did not elicit typical phenotypes of WAT browning (Figure 6D–H). Collectively, these observations suggested that activation of the DMH^GABA^ neurons to the LPO circuit may elicit white adipose browning; this circuit may mediate the function of DMH^GABA^ neurons in the central regulation of WAT browning.

### 2.7 DMH^GABA^ Neurons Induce WAT Browning through GABAergic DMH to LPO Projection

**Figure 7:**
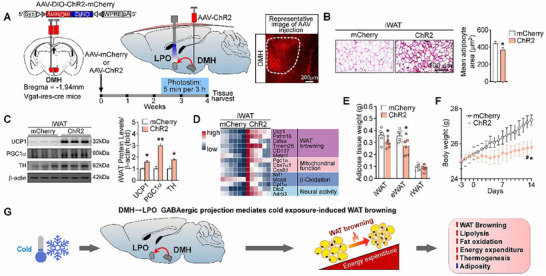
Optogenetic stimulation of DMH^GABA^ to LPO projection elicits WAT browning. A) Schematic view showing optogenetic stimulation of axonal terminals projected from DMH GABAergic neurons in the LPO. Light pattern: 5 min, 20 Hz, repeated every 3 h for 14 days. B) Representative images of iWAT H&E staining and quantification of adipocyte areas (*n* = 3 mice per group). C–F) Optogenetic activation of DMH GABAergic to LPO projection enhances WAT browning‐related protein levels (C) and gene expressions (D), reduces fat mass weights (E), and lowers body weight (F) (*n* = 6 for each group, from two independent experiments). G) Diagram illustrating the DMH GABAergic projection to LPO governs WAT browning. All data are shown as means ± SEM. The *P* values are calculated based on an unpaired Student's t‐test (B and E) and repeated measures two‐way ANOVA with Bonferroni's corrections (F). ^*^ *p* < 0.05, ^#^ *p* < 0.1.

To validate whether activation of this projection from DMH^GABA^ neurons to the LPO may lead to WAT browning, we selectively photoactivated the LPO in Vgat‐ires‐Cre mice after injection of AAV‐DIO‐ChR2:mCherry into the DMH (**Figure** **7**A). We found that the photostimulation substantially increased the number of beige adipocytes in iWAT (Figure 7B), enhanced the expression level of both proteins and genes involved in the process of WAT browning induction (Figure 7C,D), reduced fat mass weights (Figure 7E), and body weight (Figure 7F). Taken together, these results indicate that activation of the DMH^GABA^ neurons to the LPO circuit may elicit white adipose browning; this circuit may mediate the function of DMH^GABA^ neurons in central regulation of WAT browning (Figure 7G).

## 3 Discussion

It is well recognized that chronic cold exposure is one of the most potent physiological inducers of WAT browning.^[^ ^28^, ^29^, ^30^, ^31^ ^]^ Cold therapy is also an achievable lifestyle intervention that effectively improves metabolic status in humans.^[^ ^10^ ^]^ Cold exposure promotes WAT browning by activating the hypothalamus.^[^ ^32^, ^33^ ^]^ The hypothalamus, as a key brain site for integrating signals of metabolism‐related external physical stimulation, has also been recognized as a region that is involved in the central regulation of WAT browning.^[^ ^19^ ^]^ Nevertheless, the mechanism by which the hypothalamus responds to WAT browning‐inducing conditions, cold exposure, remains incompletely understood.

The DMH, a hub for integrating external stress signals and coordinating sympathetic outputs, plays central roles in the neural regulation of metabolic homeostasis.^[^ ^34^ ^]^ The DMH harbors both GABAergic and glutamatergic neurons. The GABAergic neurons are the dominant neuronal population in the DMH.^[^ ^21^, ^25^ ^]^ Activation of DMH^GABA^ neurons leads to increased EE and decreased body weight.^[^ ^32^ ^]^ Activation of bombesin receptor subtype‐3 (Brs3)‐expressing neurons in the DMH, which could be partially GABAergic neurons, also elevates EE.^[^ ^35^ ^]^ Knockdown of NPY in the DMH neurons promotes the development of beige adipocytes through the sympathetic‐adipose connection.^[^ ^36^ ^]^ In the present study, we found that the majority of activated neurons in the DMH are GABAergic under the WAT browning‐inducing cold exposure. GABAergic neurons in the DMH, rather than the glutamatergic neurons, are required to induce white fat browning. Taken together, we identified the DMH as a key hypothalamic nucleus responding to cold exposure. GABAergic neurons in the DMH may serve as a predominant neuronal population in centrally governing WAT browning.

The LPO is one of the sub‐nuclei of the preoptic area (POA) in the hypothalamus. The function of LPO has been largely unexplored.^[^ ^37^ ^]^ Emerging evidence shows that LPO is involved in the regulation of sleep,^[^ ^38^, ^39^, ^40^ ^]^ drinking,^[^ ^41^ ^]^ thermogenesis, and drug addiction.^[^ ^37^, ^42^, ^43^ ^]^ There is a connection between LPO and DMH in responses to threat stimuli, and it may play roles in sensory‐motor integration and an animal's survival.^[^ ^44^ ^]^ In the present study, we found that activation of axon terminals of DMH^GABA^ neurons in the LPO could induce typical WAT browning phenotypes, decreased adiposity, and lowered body weight. These findings demonstrate that GABAergic DMH to LPO projection may be essential for mediating cold‐induced WAT browning. The LPO may be a key site that relays the signals from the DMH for centrally regulating WAT browning.

Of note, previous studies have demonstrated that iWAT exhibits markedly higher sympathetic innervation density, enhanced vascularization, and superior browning potential compared to eWAT.^[^ ^45^, ^46^ ^]^ iWAT adipocytes display a more pronounced transcriptional response to β‐adrenergic stimulation, characterized by robust upregulation of thermogenic genes such as Ucp1 and Pgc1α. In contrast, eWAT remains largely unresponsive to thermogenic stimuli, likely due to its limited sympathetic input and its predominant function as a site for energy storage rather than thermogenesis.^[^ ^45^, ^47^ ^]^

Although our findings demonstrate that activation of DMH GABAergic neurons promotes thermogenic remodeling in rodent adipose tissue, caution is warranted when extrapolating these results to primates or humans. In rodents, particularly mice, white adipose depots such as iWAT retain considerable browning plasticity and are richly innervated by sympathetic fibers, which enables robust thermogenic responses to cold and central stimulation.^[^ ^1^, ^48^ ^]^ In contrast, adult humans exhibit a much more limited capacity for WAT browning, with thermogenic activity primarily restricted to classical brown fat depots such as the supraclavicular region.^[^ ^49^, ^50^ ^]^ Furthermore, compensatory behaviors, such as increased energy intake, often blunt the long‐term metabolic benefits of cold exposure in humans, rendering its anti‐obesity effects controversial and difficult to sustain.^[^ ^51^, ^52^ ^]^ To date, whether DMH GABAergic neurons are involved in the central regulation of WAT browning in primates remains to be explored. Functional neuroimaging studies reveal that the human hypothalamus is responsive to cold,^[^ ^14^, ^16^, ^53^, ^54^, ^55^ ^]^ and the cellular and circuit‐level mechanisms, particularly within the dorsomedial region, demand investigation. Together, these explorations uncovered that deciphering the hypothalamic mechanism underlying WAT browning in rodents is an essential step for improving the metabolic health of humans in the future.

In summary, we identified that the DMH may serve as a key nucleus that responds to WAT browning‐inducing cold exposure. The GABAergic neurons in the DMH may act as an important neuronal population that regulates cold‐induced WAT browning. We uncovered a previously unknown neural circuitry: a GABAergic projection from the DMH to the LPO, which may mediate the induction of WAT browning. Identification of neural structure controlling cold‐inducing WAT browning may provide new insights into the mechanisms of central regulation of energy homeostasis, as well as the anti‐obesity and health‐promoting effects of cold therapy.

## Funding

This work was supported by grants from the Noncommunicable Chronic Diseases‐National Science and Technology Major Project (2024ZD0530200, 2024ZD0530201 to R.Z., 2024YFA1107500 to W.S.), the National Natural Science Foundation of China (82170864, 81471064, 81670779, and 81870590 to R.Z.), the National Key Research and Development Program of China (2017YFC1700402 to R.Z.), the Beijing Municipal Natural Science Foundation (7162097 and H2018206641 to R.Z.), the Peking University Research Foundation (BMU20140366 to R.Z.), and the Scientific Project of Beijing Life Science Academy (2023300CB0100 to R.Z.), National Natural Science Foundation of China (No. 32300849 to Q.Z.; No. 32330042, No. 32425028, and No. 92357304 to W.S.), Scientific and Technological Innovation 2030 (2025ZD0216300 to W.S.), Strategic Priority Research Program of the CAS (XDB1010000 to W.S.).The authors thank the Shanghai Municipal Government and ShanghaiTech, and Shanghai Frontiers Science Center for Biomacromolecules and Precision Medicine University for financial support.

## Conflict of Interest

The authors declare no conflict of interest.

## Supporting information



Supporting Information

## Data Availability

The data that support the findings of this study are available from the corresponding author upon reasonable request.
