# Supplementary material for: The DMHGABA Neurons Play a Crucial Role in the Regulation of Cold‐Induced White Adipose Browning through DMH to LPO Projection
Source: Adv Sci (Weinh). 2025 Nov 5;13(5):e08513. doi: 10.1002/advs.202508513 (PMC12850182; doi:10.1002/advs.202508513)

**The DMH^GABA^ Neurons Play A Crucial Role in The Regulation of Cold-Induced White Adipose Browning Through DMH to LPO Projection**

Zhijie Su^1^†, Yujia Hou^1^†, Bingwei Wang^2^†, Qian Zhou^3^†, Wen Yang^3^, Bingbing Guo^1^, Chenyu Zhang^1^, Miao Zhao^1^, Xiaoning Yang^1^, Jiarui Liu^1^, Xiangyang Xie^4^, Lihua Qin^1^, Weiguang Zhang^1^, Wei L. Shen^3^*, Ruimao Zheng^1,5,6,7,8^ *

**Fig. S1. Cold exposure increases food intake, promotes sympathetic activation of iWAT, and induces widespread c-Fos expression in multiple brain regions.**

(**A**) Daily food intake (g/mouse/day) under room temperature or cold exposure conditions. The P values are calculated based on unpaired Student’s t-test; n = 6 mice/group. (**B**) Representative immunofluorescence images and quantification of TH staining in iWAT under RT and cold exposure. TH signal (green) was quantified as % intensity relative to RT control. The P values are calculated based on unpaired Student’s t-test; n = 4 mice/group. (**C**) Summary table of c-Fos immunoreactivity in brain regions involved in thermoregulation following cold exposure. The intensity of c-Fos-positive neurons was semi-quantitatively scored from ‘+’ (low) to ‘++++’ (very high) based on staining density. **P* < 0.05, ***P* < 0.01.

**Number of c-Fos+ neurons in brain regions under cold exposure.**

**Fig. S2. Ablation of DMH^Vgat^ neurons leads to adiposity.**

(**A**) Scheme for disrupting GABA release in DMH neurons by AAV. (**B**) Body weight of GFP (n = 5) and Cre mice (n = 4). (**C**) The percentage of fat mass of GFP (n = 5) and Cre mice (n = 4). (**D**) Food intake of GFP (n = 5) and Cre mice (n = 4). (**E**) Intraperitoneal Glucose Tolerance Test. Blood glucose was measured at the indicated time points after intraperitoneal glucose injections (GFP: 8 mice; Cre: 11 mice). (**F**) Locomotor activity was measured in GFP control mice (n = 5) and DMH^Vgat^ Cre ablation mice (n = 4). All data are shown as means ± SEM. The *P* values are calculated based on two-way ANOVA analyses followed by Bonferroni’s post hoc test (B, D and E) or unpaired Student’s t-test (C and F). **P* < 0.05, ****P* < 0.001.

**Fig. S3. Effects of DREADD induced DMH^Vgat^ neuron activation on WAT browning and energy metabolism, related to Fig. 5.**

(A) c-Fos immunoactivity induced by chemogenetic activation of DMH GABAergic neurons. The CNO was delivered by i.p. injection. Representative images were from three independent experiments (n = 3 mice per group). White arrows indicate neurons that were double-positive for c-Fos (green) and hM3Dq (red). (B) Representative images of H&E staining and quantification of adipocyte areas in the eWAT after chemogenetic activation (hM_3_Dq) or inhibition (hM_4_Di) of DMH neurons in Vgat-ires-Cre or Vglut2-ires-Cre mice. Scale bar: 100 μm. (**C**) Quantification of average daily food intake under different DREADD manipulations in both Vgat- and Vglut2-ires-Cre mice. **(D–F)** Indirect calorimetry analysis of oxygen consumption (VO₂), carbon dioxide production (VCO₂), and respiratory exchange ratio (RER) in Vgat-ires-Cre mice expressing mCherry, hM_3_Dq, or hM_4_Di. **(G)** Locomotor activity in Vgat-ires-Cre mice across light and dark phases. **(H–J)** Indirect calorimetry analysis of VO₂, VCO₂, and RER in Vglut2-ires-Cre mice. **(G)** Locomotor activity in Vglut-ires-Cre mice across light and dark phases. All data are shown as means ± SEM. Statistical analyses in (B, C, G and F) were performed using repeated measures two-way ANOVA with Tukey’s corrections. Statistical analyses in (D-F and H-J) were performed using covariance (ANCOVA) to determine statistical differences for in vivo metabolic analyses. *P < 0.05, ***P < 0.001.

**Fig. S4. Optogenetic activation of DMH^Vgat^ neuron promotes WAT browning.**

(**A**) Schematic illustration of virus injection and photostimulation protocol. AAV-DIO-ChR2 or AAV-DIO-mCherry was bilaterally injected into the DMH of Vgat-ires-Cre mice, followed by optic fiber implantation and 470 nm photostimulation (5 min every 3 h) for 2 weeks. (**B**) Representative thermal images and quantification of iWAT surface temperature in mCherry and ChR2 groups. (**C**) Representative thermal images and quantification of BAT and tail surface temperature. (**D**) Relative mRNA expression levels of Ucp1 and Pgc1α in iWAT measured by qPCR. (**E**) Representative H&E-stained sections and quantification of mean adipocyte size in eWAT. n = 6/group. Data are presented as mean ± SEM. The P values are calculated by using unpaired Student’s t-test (B-E). *P < 0.05, **P < 0.01, ***P < 0.001.

**Fig. S5. Optogenetic activation of DMH^Vgat^→LPO projections induces WAT browning.**

(**A**) Schematic of the experimental design. **(B)** Representative thermal images and quantification of iWAT surface temperature in mCherry and ChR2 groups. (**C**) Representative thermal images and quantification of BAT surface temperature. Data are presented as mean ± SEM. The P values are calculated by using unpaired Student’s t-test (B, C). **P < 0.01.

**Fig. S6. Original western blot images of this manuscript.**

**Fig. S7. Original western blot images of this manuscript.**

**Fig. S8. Primers used in this study.**

**Materials and Methods**

**Mice**

Animal studies were approved by the Institutional Care and Use Committee of the Peking University Health Science Center and the Animal Care and Use Committee of ShanghaiTech University, Shanghai Model Organisms Center, Inc., and the Animal Facility at the National Facility for Protein Science in Shanghai (NFPS), Zhangjiang Lab, China. All experiments were performed on male adult mice (8 to 12 weeks old). Mice were housed at 22 ± 1 °C with a 12-hours light/dark cycle (light time, 20:00 to 8:00) with ad libitum chow food (4% fat SPF rodent feed) and water. The following mice strains were from The Jackson Laboratory: C57BL/6J Vgat-ires-cre knock-in mice (Jax 028862), C57BL/6J Vglut2-ires-cre knock-in mice (Jax 028863), Vgat^flox^ (Jax no. 012897) and C57BL/6J (Jax 000664). The Vgat-ires-Cre mice were crossed with Cre-dependent Gt (ROSA) 26Sortm14(CAG-tdTomato)Hze/J mice (Ai14, Jax 007908) to reveal GABAergic expression patterns. All experimental protocols were approved by the Animal Care and Use Committee of the Peking University Health Science Center, and the Animal Care and Use Committee of ShanghaiTech University, following institutional guidelines for the care and use of animals. Body weights were continuously measured for all animals before sacrifice.

**Reagents**

CNO (#C0832, Sigma-Aldrich); optimal cutting temperature (OCT) compound (Tissue-Tek); rabbit anti-cFos (Synaptic systems, #226003); rabbit anti-UCP1 (Abcam, ab10983); rabbit anti-Pgc1α (Millipore, AB3242); rabbit anti-TH (Millipore, AB152); mouse anti-β-actin (Sigma-Aldrich, A5316); HRP AffiniPure Goat Anti-Rabbit IgG(H+L) (1:5000, Biodragon, BF03008); HRP AffiniPure Goat Anti-Mouse IgG(H+L) (1:5000, Biodragon, BF03001); phosphatase inhibitors (B15002, Bimake), and protease inhibitor cocktail (B14002, Bimake). Tranzol up, TransScript one-step gDNA removel and cDNA synthesis Super MiX, TransStart Top Green qPCR Super Mix were purchased from TransGen Biotech (Beijing, China). RNaseZapTM were purphased from Invitrogen (Beijing, China).

**Cold exposure protocol**

The long-term cold exposure was conducted according to a previously established program. In brief, 10-week-old mice were housed individually with ad libitum food and water. Mice were placed at 4 ℃ for seven days before sacrificing. After sacrifice, various tissue samples were collected as indicated in the figure and frozen in liquid nitrogen for RNA and protein analysis or fixed in 1× PBS buffer containing 4% PFA for histology. Some mice were perfused, their brains processed for immunohistochemistry and neurons scored for c-Fos expression.

**Stereotaxic injections and optic fiber implantation**

Mice were anesthetized with pentobarbital (80 mg/kg; i.p.). During surgery, mice were held in a stereotaxic holder and the skull was exposed via a small scalp incision74. The craniotomy was performed using a 0.6-mm drill tip. Viruses were bilaterally injected into the DMH (coordinate, posterior to bregma, AP; lateral to the midline, ML; below the brain surface, DV): AP -1.55 mm, ML ± 0.35 mm, DV -5.30 mm.

To block DMH neurons, AAV2/9-hSyn-DIO-GFP: TetTox (0.3 μl per injection site) was bilaterally injected into the DMH of Vgat-ires-Cre mice or Vglut2-ires-Cre mice as indicated. Mice were recovered in a warm bracket before being transferred to housing cages for two weeks. Then mice received cold exposure.

For the chemogenetic activation or inhibition of DMH neurons, AAV2/9-hSyn-DIO-hM3Dq-mCherry or AAV2/9-hSyn-DIO-hM4Di-mCherry (0.3 μl per injection site) was bilaterally injected into the DMH of indicated genotypes, respectively. Mice were recovered in a warm bracket before being transferred to housing cages for two weeks. Then mice received twice-daily intraperitoneal injections of CNO (0.3 mg/kg) to modulate neural activity for seven days at room temperature.

To knockout the VGAT gene in DMH neurons, AAV2/9-hSyn-cre-t2a-GFP or AAV2/9-hSyn-GFP (0.15 μl per injection site) was bilaterally injected into the DMH of Vgat^loxp/loxp^ mice. Mice were allowed to recover in a warm blanket before they were transferred to housing cages for 2-4 weeks before performing evaluations.

To locally stimulate DMH neural terminals at different brain sites, we injected AAV2/9-hSyn-DIO-hM3Dq-mCherry (0.3 μl per injection site) bilaterally into the DMH of Vgat-ires-Cre mice. After two weeks of recovery, brain infusion cannulas were implanted in the LPO (AP -0.26 mm, ML ± 0.75 mm, DV -4.75 mm), the PVT (AP -0.34 mm, ML 0.0 mm, DV -2.70 mm), the LPAG (AP -4.8 mm, ML ± 0.6 mm, DV -1.9 mm) and the LPBN (AP -5.34 mm, ML ± 1.35 mm, DV -2.60 mm). After recovery for seven days, we twice-daily infused CNO (0.5 mM, 0.3 μl per injection site) for a week, via the cannula to stimulate neural terminals, under briefly isoflurane anesthesia.

For optogenetic experiments, AAV2/9-hSyn-DIO-ChR2-mCherry vectors (0.3 μl per injection site) was delivered bilaterally into the DMH of Vgat-ires-Cre mice. Then, the fiber-optic cannula was inserted into the DMH (200 µm in diameter, NA 0.22; coordinates, AP -1.55 mm, ML 0.35 mm, DV -5.10 mm) or the LPO (AP -0.26 mm, ML 0.75 mm, DV -4.55 mm) and fixed on the skull with dental cement. After two weeks of recovery, mice were photostimulated for 5 min (470 nm, 20 Hz, 5 mW) every 3 hours, with the simulation repeated for 14 days.

**Immunocytochemistry**

Mice were perfused transcardially with phosphate-buffered saline (1× PBS), followed by 1× PBS buffer containing 4% PFA. For c-Fos staining, animals were perfused and fixed immediately after the end of cold exposure. Brains were dissected out and post-fixed overnight at 4 ℃ by 4% PFA in 1× PBS, followed by cryoprotection in 1× PBS containing 20-30% gradient sucrose solution (wt/vol) at 4 ℃. Free-floating sections (30 μm) were prepared with a microtome (Leica). Brain slices were incubated with primary antibodies, rabbit anti-cFos (1:20, 000), for 24 hours at 4 ℃, followed by 3× washes in PBST (PBS with 0.1% Triton X-100, vol/vol) before incubation in secondary antibodies (Alexa-Fluor 488-conjugated secondary antibodies, 1:500) for 2 hours at room temperature. Antibodies were incubated in blocking buffer (1× PBS containing 2.5% normal goat serum (vol/vol), 1.5% Bovine serum albumin (weight/vol), 0.1% Triton™ X-100 (vol/vol)). For each animal, coronal brain sections (thickness: 30 μm) were collected. Every other section was selected for analysis to ensure coverage of the region of interest. c-Fos⁺ cells were counted bilaterally within anatomically defined boundaries based on the mouse brain atlas. Quantification was performed by two independent investigators blinded to group identity. For each animal, the final value represents the average number of c-Fos⁺ cells across all analyzed sections. Images were captured on a Leica microscope or Olympus VS120 Virtual Microscopy Slide Scanning System.

**Histological analysis**

Mice were perfused transcardially with 1× PBS, followed by 1× PBS buffer containing 4% PFA. The pieces of freshly dissected iWAT and eWAT samples were fixed by 4% PFA in 1× PBS for 24 hours, followed by cryopreservation in 1× PBS containing 20-30% gradient sucrose solution (wt/vol) overnight and before embedding in OCT compound. Samples were sectioned and stained with H&E for further morphological investigation by a standard procedure. To measure lipid droplet sizes, Images were captured on a Leica microscope (DMI 4000B, Wetzlar, Germany) or Vectra Polaris Automated Quantitative Pathology Imaging System (Perkin Elmer). An analyst blinded to experimental conditions used ImageJ software to quantify lipid droplet areas in at least 200 adipocytes per mouse (n = 3 mice per group, with 3 - 6 sections per mouse).

**Quantitative PCR**

The total RNA was extracted from inguinal adipose tissue using the TRIzol reagent and determined the quality and quantity using a NanoDrop (Thermo Fisher Scientific). Then, the mRNA was reverse transcribed using a High-Capacity cDNA Reverse Transcription Kit according to the manufacturer’s instructions and processed for quantitative real-time PCR using the SYBR Green PCR system (Bio-Rad, Hercules, CA, USA). Relative mRNA expression was determined by normalization with the expression of genes of interest using the comparative Ct method. The GAPDH used as an endogenous control. The primer sequences used in this study were listed in Figure S6. For brains, cold exposure, running exercise, swimming exercise or control mice were perfused briefly with sterile PBS, brains were dissected out quickly and the DMH region was microdissected under a dissection scope. RNA was isolated from the sample using TRIzol reagent following the manufacturer’s protocol.

**Western blotting analysis**

Tissues were homogenized in a RIPA lysis buffer containing 0.5% NP-40, 0.1% sodium deoxycholate, 150 mM NaCl, 50 mM Tris-HCl (pH 7.4), phosphatase inhibitors, and protease inhibitor cocktail. Tissue extracts were then immunoblotted with the following primary antibodies: rabbit anti-UCP1 (1:1000); rabbit anti-Pgc1α (1:1000); rabbit anti-TH (1:1000,); mouse anti-β-actin (1:2000); and secondary antibodies: HRP AffiniPure Goat Anti-Rat IgG(H+L) (1:5000); HRP AffiniPure Goat Anti-Mouse IgG(H+L) (1:5000). The blots were developed using an Enhanced Chemiluminescence assay (Bio-Rad, Hercules, CA, USA). We analyzed the band density of western blot images using the ImageJ software.

**Metabolic measurement**

For chemogenetic experiments, on day 6 of CNO treatment, the mice were placed in metabolic chambers with fresh food and water to acclimate for 24 hours. On day 7, CNO (0.3 mg/kg) was administered within 90 minutes before the dark cycle and EE was monitored (LE1305 Physiocage 00; LE405 O2/CO2 Analyzer; LE400 Air Supply and Swithching). We used Metabolism v2.2.01 to analyze the data.

**Body weight and body composition analyses**

Body weight was continuously measured for all animals before mice sacrificing. After 4 weeks viral expression, body composition was determined using a Minispec whole-body composition analyzer (Burker Minispec CMR LF50 ).

**Glucose tolerance test**

For ipGTT, fasting blood glucose was measured after 16-h fasting. Then mice were intraperitoneally injected glucose (2.0 g/kg). Blood glucose was measured at 0, 15, 30, 60 and 120 min using a hand-held glucometer (Accu-Chek Performa Connect, Roche, Switzerland).

**Activity monitoring**

The locomotor activity was monitored by the Comprehensive Lab Animal Monitoring System with Temperature Telemetry Transmitter (CLAMS; G2 E-Mitter).

**RNA-sequence analysis**

The total RNA from the DMH was extracted using TRIzol reagent. A total amount of 3 μg RNA per sample was used as input material for the RNA sample preparations. After that, the RNAs were subject to 50-bp single-end sequencing with a BGISEQ-500 sequencer. At least 20 million clean reads of sequencing depth were obtained for each sample. The DESeq R package (1.10.1) was used to perform the differential expression analysis in digital gene expression data using a model based on the negative binomial distribution. The resulting P-values were adjusted using Benjamini and Hochberg’s approach for controlling the false discovery rate. Genes with an adjusted P-value < 0.05 by DESeq were assigned as differentially expressed. DEGs were defined as genes with FDR less than 0.01 and log2 fold change larger than 1 (upregulation) or smaller than -1 (downregulation). GO and pathway annotation and enrichment analyses were based on the Gene Ontology Database (http://www.geneontology.org/) and KEGG pathway database (http://www.genome.jp/kegg/), respectively. The GO and KEGG terms presented in Figure 2C and 2D represent the top-ranking enriched terms based on adjusted P value (Q value). To ensure biological relevance, we specifically selected the top 10 terms related to neural and metabolic processes for visualization.

**Statistical analysis**

The statistical significance was determined with SPSS (Windows version 26) or GraphPad Prism software (version 8.0; Origin Laboratory). Data distribution was assessed using the Kolmogorov-Smirnov test. As indicated, statistical significance was determined using Student’s t-test, one-way or two-way ANOVA, and then either Tukey’s or Bonferroni’s multiple comparison test to compare all treatment groups. Sample sizes, statistical tests, and P values were indicated in the text, figures, and figure legends. A P-value less than 0.05 was considered statistically significant. Data are presented as the mean ± SEM. *P < 0.05, **P < 0.01, ***P < 0.001.


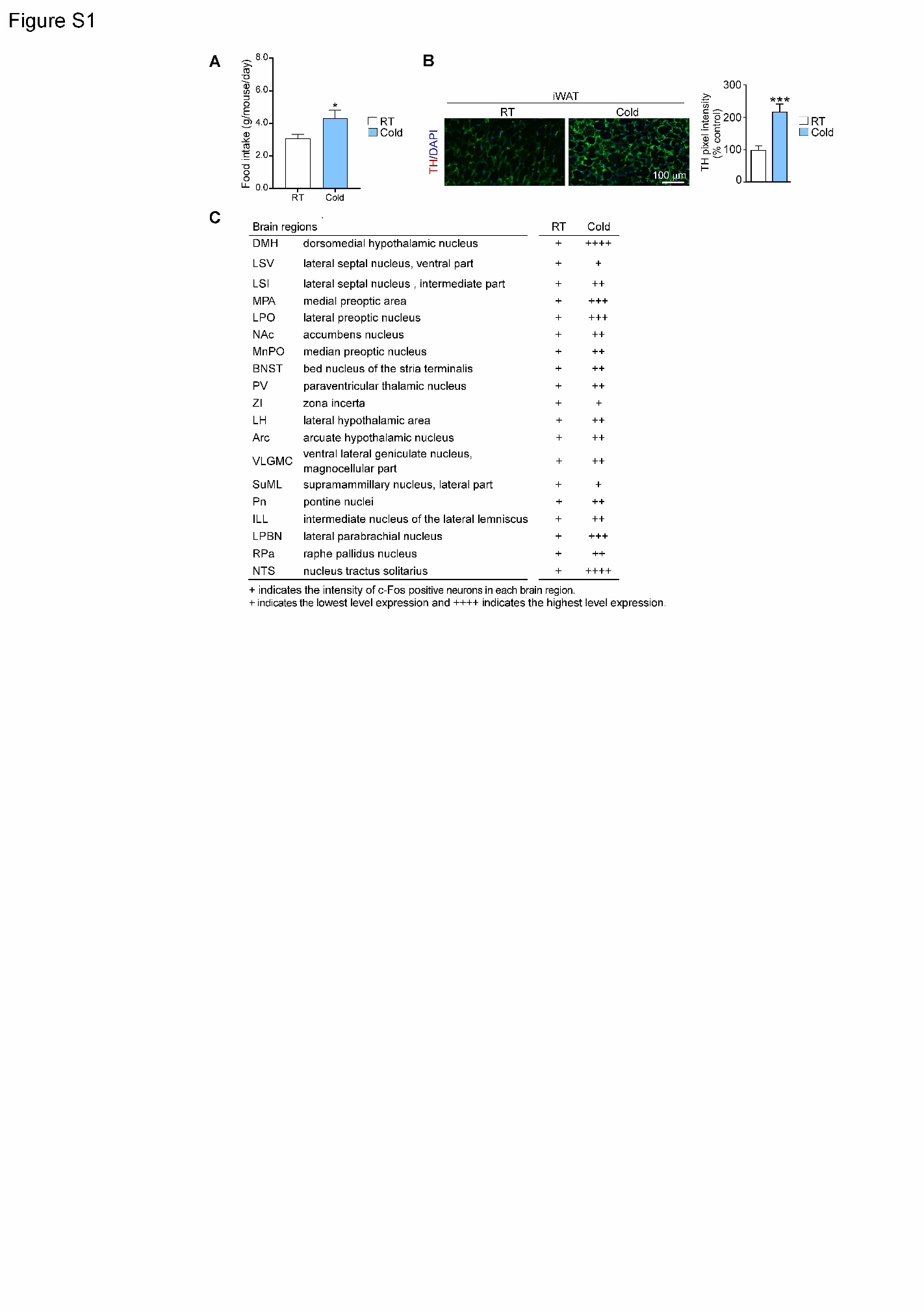


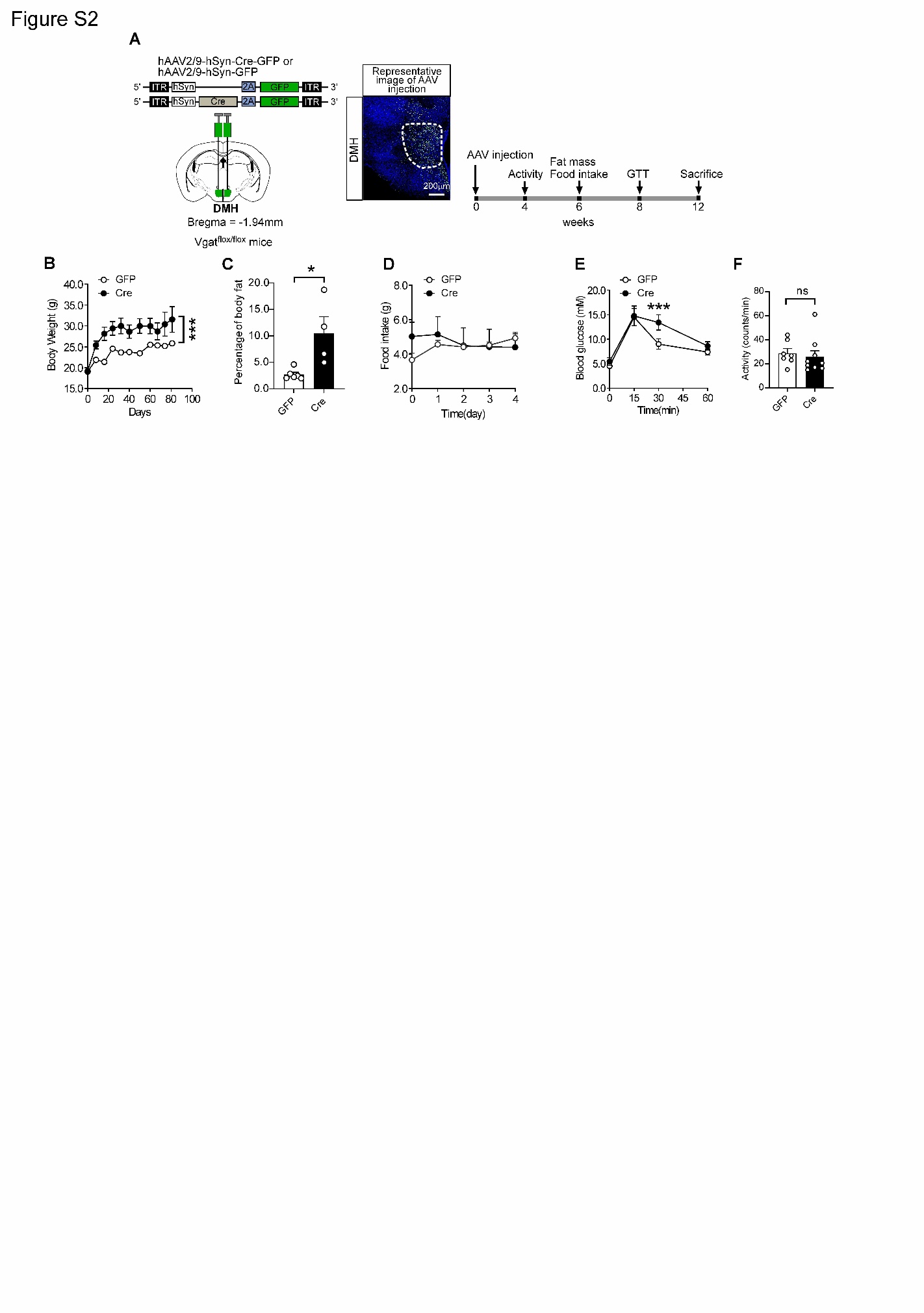


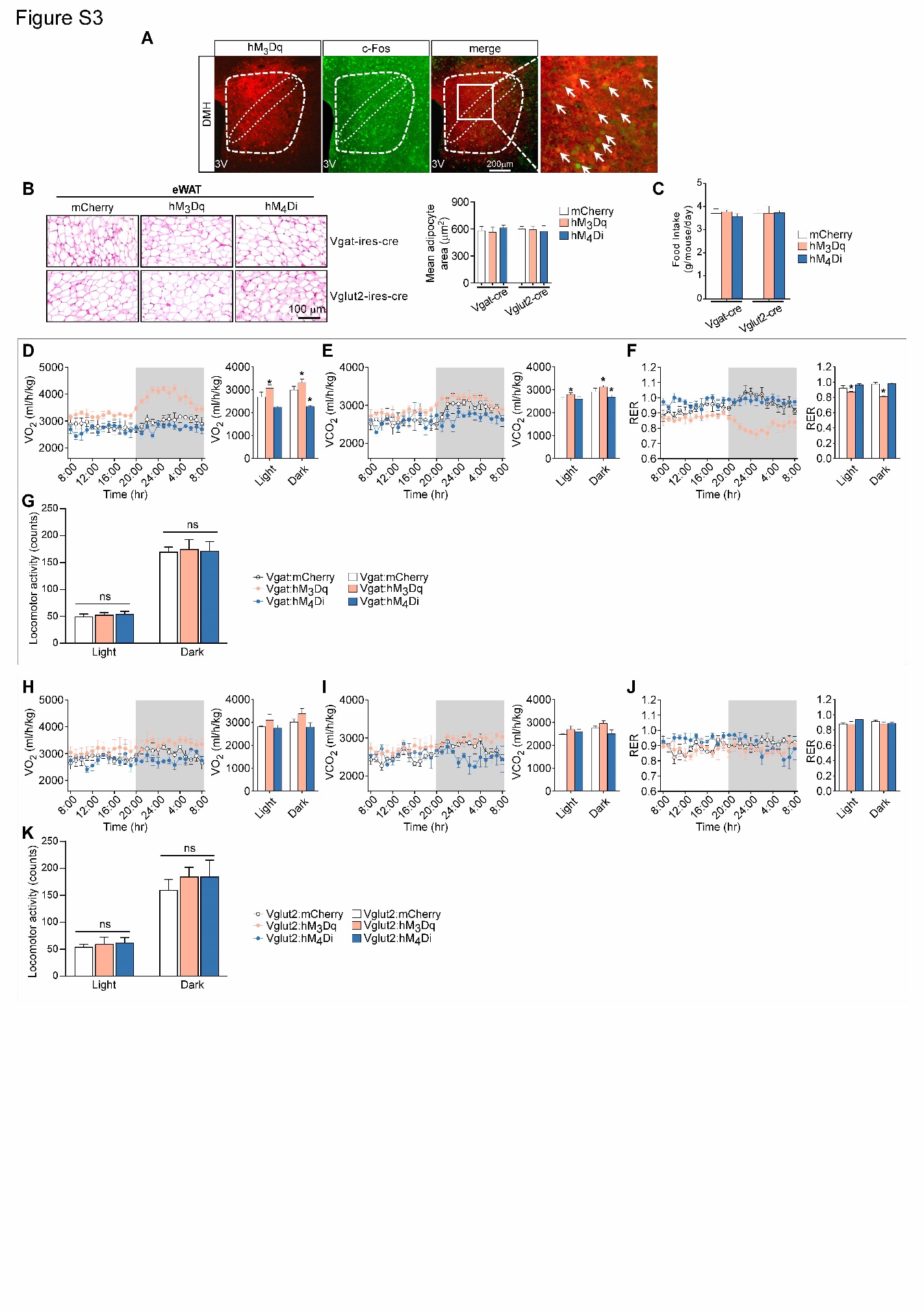


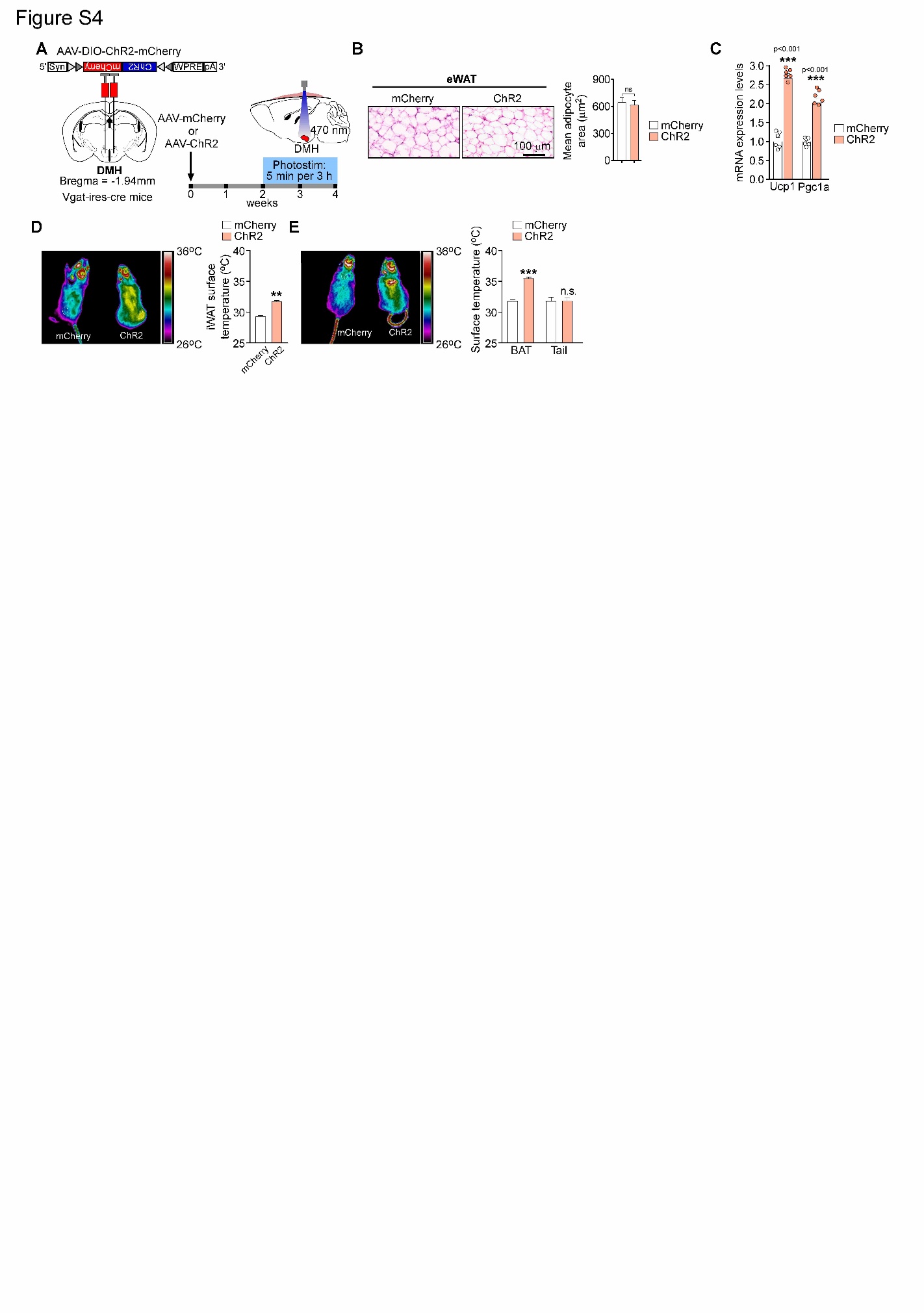


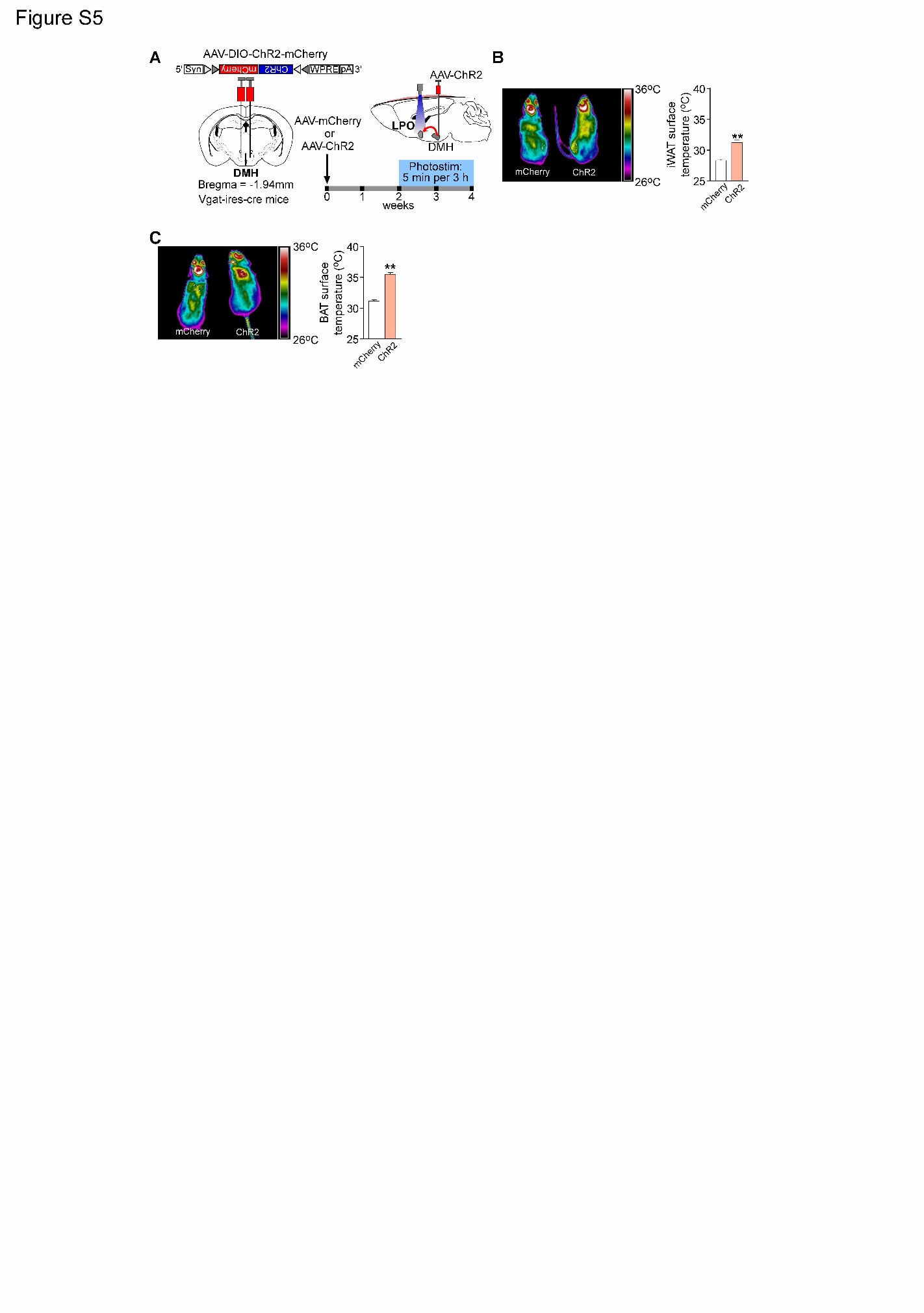


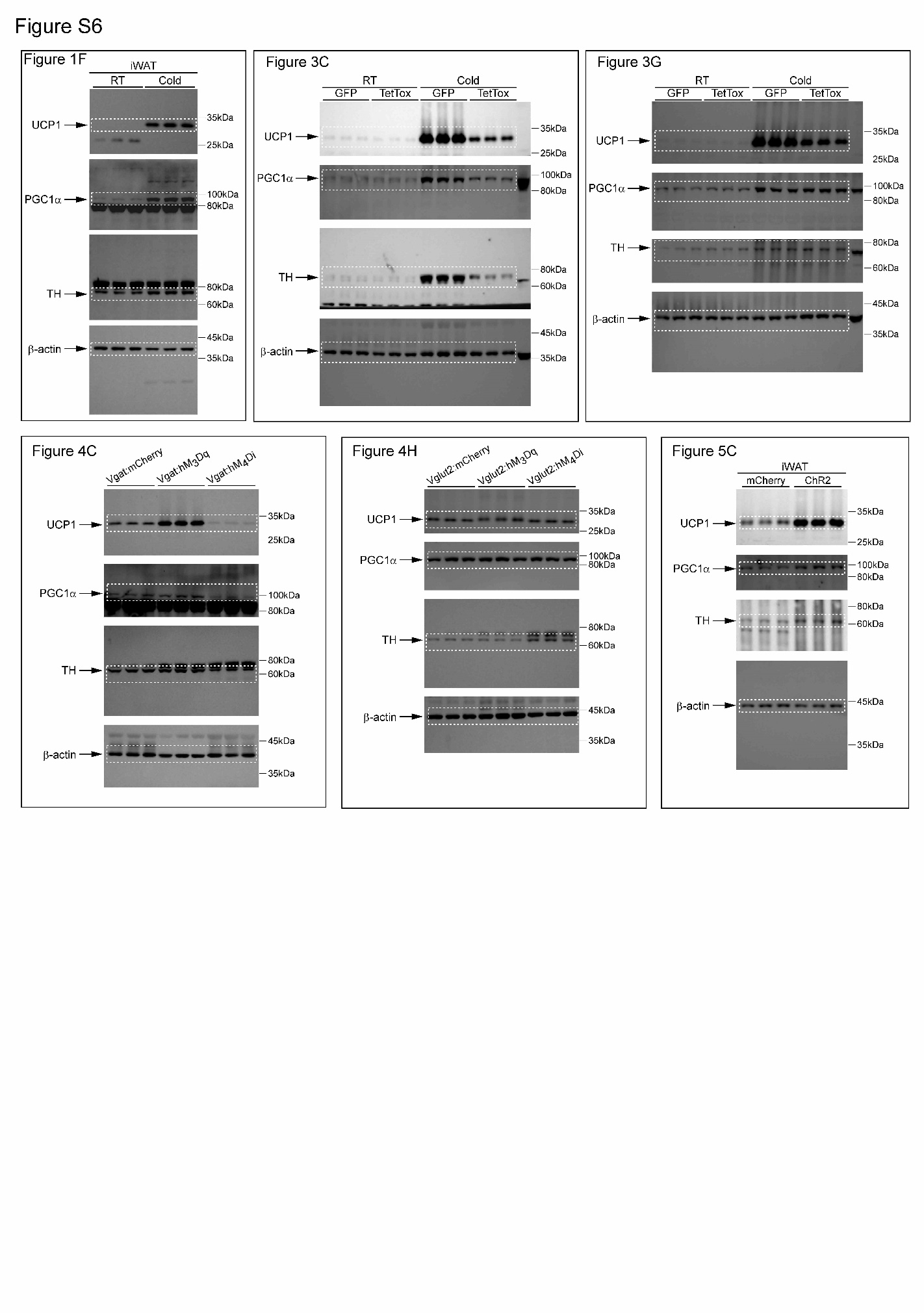


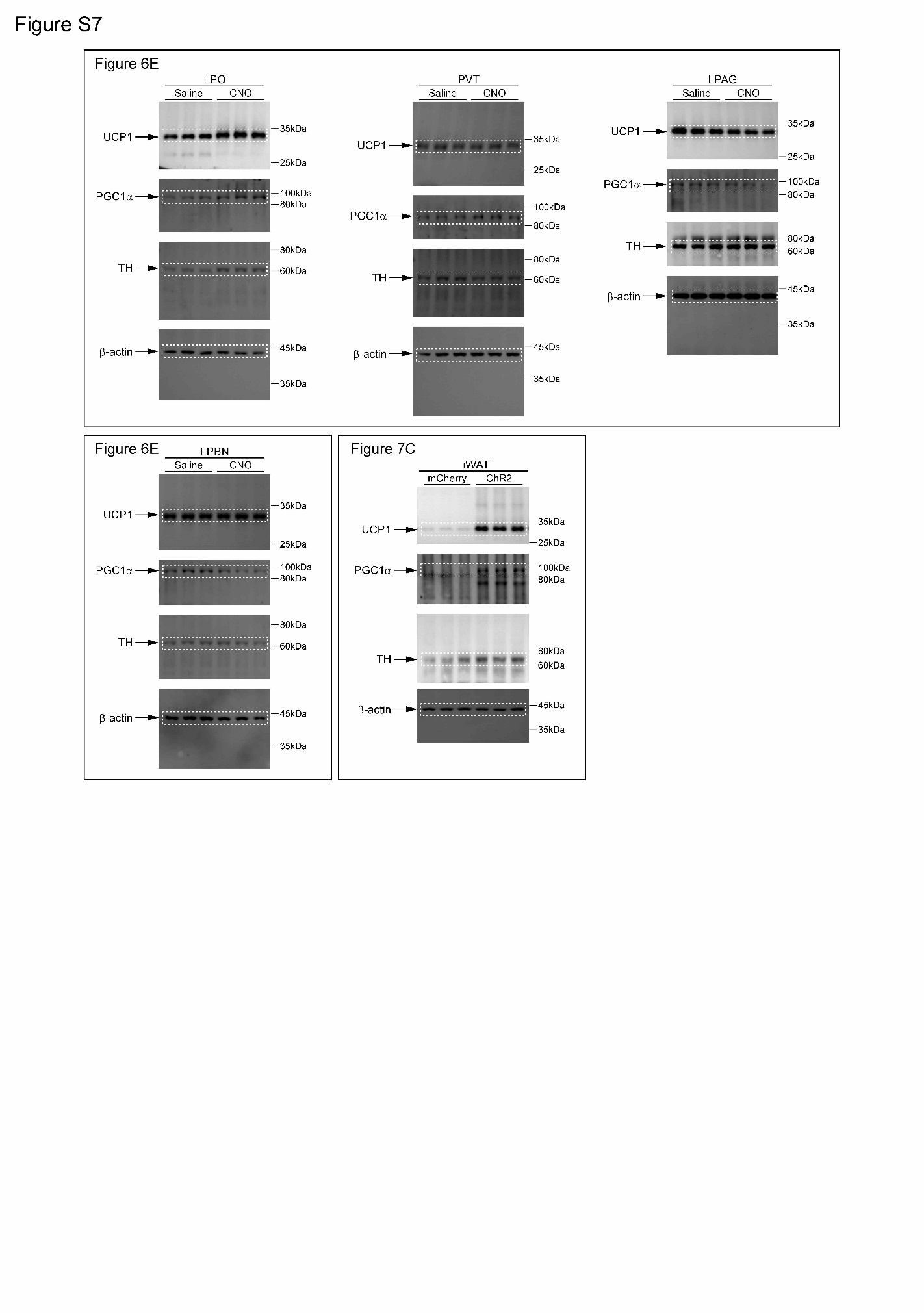


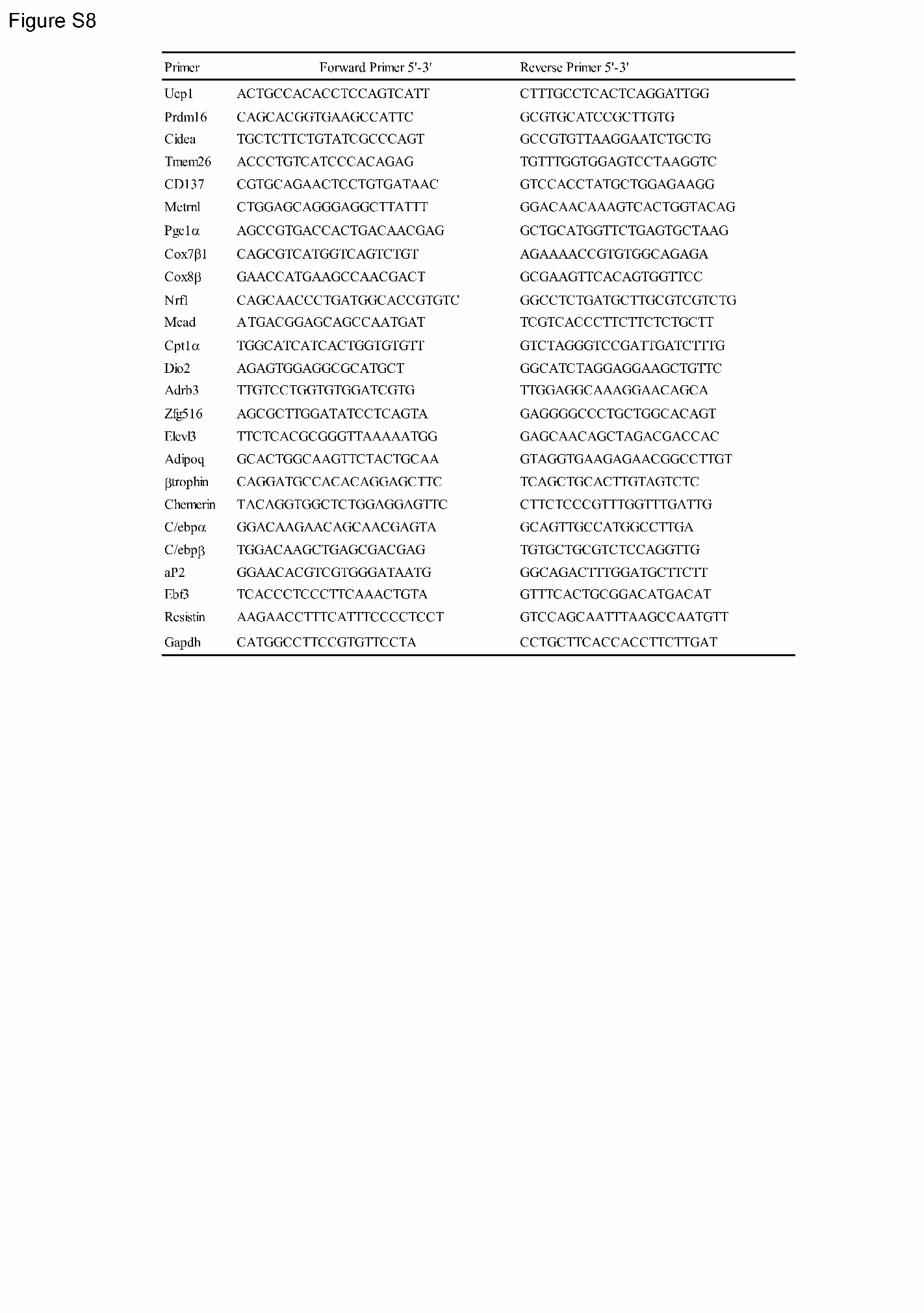

Supplement: Supplementary file 1 — Supporting Information [file ADVS-13-e08513-s001.docx]
